# Agreement between retrospectively and contemporaneously collected patient-reported outcome measures (PROMs) in hip and knee replacement patients

**DOI:** 10.1007/s11136-018-1823-6

**Published:** 2018-02-26

**Authors:** Esther Kwong, Jenny Neuburger, Nick Black

**Affiliations:** 1 Department of Health Services Research, Faculty of Public Health and Policy, London School of Hygiene and Tropical Medicine, 15-17 Tavistock Place, London, WC1H 9SH UK; 2 London School of Hygiene and Tropical Medicine, London, UK

**Keywords:** Patient-reported outcome measures, Health status, Health-related quality of life, Retrospective, Recall, Agreement

## Abstract

**Purpose:**

To investigate the relationship between retrospectively and contemporaneously collected patient-reported outcome measures (PROMs) and the influence on this relationship of patients’ age and socio-economic status and the length of time.

**Methods:**

Patients undergoing hip or knee replacement in four hospitals who had completed a pre-operative questionnaire were invited to recall their pre-operative health status shortly after surgery. The questionnaires included a disease-specific (Oxford Hip Score; Oxford Knee Score) and generic (EQ-5D-3L) PROM. Consistency and absolute agreement between contemporary and retrospective reports were investigated using intraclass correlations (ICCs). Differences were visualised using Bland–Altman plots. Linear regression analysis explored whether retrospective can predict contemporary PROMs.

**Results:**

Patients’ recalled health statuses were similar to their contemporaneous reports, with no significant systematic bias. Absolute agreement for disease-specific PROMs was very strong (ICC 0.82) and stronger than for the generic PROM (ICC 0.60, 0.62). Agreement was consistently strong across the range of severity of a patient’s condition, age and socio-economic status. Patients’ age and socio-economic status had no significant influence on size of difference and direction of recall, although reliability of recall was slightly worse among the over-75s versus under-60s for hips (Oxford Hip Score ICC 0.88 vs. 0.78). Mean retrospective PROMs for groups or populations of patients can reliably predict what mean contemporary reports of PROMs would have been.

**Conclusion:**

Retrospective PROMs can be used to obtain a baseline assessment of health status when contemporary collection is not feasible or cost effective. Research is needed to determine the feasibility of retrospective PROMs in emergency admissions.

## Introduction

Patient-reported outcome measures (PROMs) have the potential to transform health care delivery through enhancing the clinical management of patients and assessing the quality of providers’ performance [1, 2]. To date, the use of PROMs in assessing the outcome of hospital admissions has inevitably been restricted to elective surgery in which before and after measurements of patients’ symptoms, functional status and health-related quality of life can be compared. The most ambitious example of this covers four elective surgical procedures in the NHS in England [3].

The challenge of using PROMs for emergency admissions, which account for 40% of hospital inpatients in England, has not been addressed and yet this is an area of increasing resource use, political importance and concern about variations in quality of care [4]. The methodological challenge is how to quantify outcome when a patient’s health status before their sudden and unexpected ill-health that led to an emergency hospital admission is, inevitably, not available. One potential solution would be if patients were able to recall accurately their health status before the admission. If they could, then a retrospective (or recalled) PROM would offer a means of obtaining their baseline health status in the absence of a prospectively collected contemporary report.

A recent literature review on the relationship between retrospective and contemporary health status reports found strong agreement when the recall period is short [5]. However, only six studies have been undertaken of which only one was conducted in the UK [6]. The relevance of findings from other countries is uncertain given the potential influence of culture and other contextual factors. In addition, only two studies considered the influence of patients’ characteristics, such as social demographic factors, on the relationship. Both studies found that agreement was slightly weaker in older patients [7, 8].

Our aim was to investigate the relationship between retrospective and contemporary PROMs in England (inevitably, in elective conditions) and to explore the influence on the relationship of two patient characteristics (age, socio-economic status) and the length of time between the two data collection points. Contemporary reports are often considered the ‘gold standard’ so if retrospective reports differ, it is the latter that are judged ‘unreliable’. However, in the context of PROMs, from a patient’s point of view the way they recall their previous health may be of greater relevance to them and to assessing the quality of health care than how they assessed it at the time. Rather than assuming one as the ‘gold standard’ over the other type of PROM, we consider the extent to which they agreed. We hypothesise that if the two agree then one can substitute for the other without any impact on assessment of the impact of health care interventions. If they differ, it would be necessary to consider the reasons for this and its implications for the use of PROMs in clinical management and in provider comparisons in emergency admissions.

## Methods

### Sample

This is a multi-centre study of patients undergoing either hip or knee arthroplasty (primary operation or revision surgery) in four hospitals, which were part of the North Thames Academic Health Science Network (UCL Partners), and CLAHRC. Health Research Authority ethics approval was obtained from North East – Newcastle & North Tyneside 2 Research Ethics Committee (REC Ref: 16/NE/0081).

Patients were eligible if, as part of the National PROMs Programme, they had completed a PROM questionnaire before undergoing surgery (Q1), either at a pre-operative assessment clinic or on their day of admission. They were invited to complete a retrospective PROM questionnaire (QR) in the immediate post-operative period prior to discharge asking them to recall their health status during the 4 weeks prior to surgery. Written informed consent was obtained.

Patients’ QR was deterministically linked to their contemporaneous PROMs data (Q1) using a hierarchy of patient identifiers: NHS number, date of birth, postcode and date of birth and postcode combined.

### Questionnaires

The self-reported questionnaires included socio-demographic information: age; sex; living arrangement (with family/friends, alone, other). Socio-economic status (SES) was measured with national quintiles of the Index of Multiple Deprivation based on patients’ residential postcode [9]. Self-reported health included co-morbidities (from a list of 12 conditions); duration of primary condition (< 1, 1–5, 6–10, > 10 years); primary or revision surgery; disease-specific PROM (Oxford Hip Score or Oxford Knee Score); and a generic PROM (EQ-5D-3L)—the latter was used as it was the version used in the National PROMs Programme in England for elective surgery at the time.

The Oxford Hip Score (OHS) is a disease-specific PROM for patients undergoing total hip replacement to capture symptoms and functional status [10]. It has good face validity, construct validity and reliability, and is sensitive to change. The Oxford Knee Score (OKS) is the knee arthroplasty equivalent [11]. For both PROMs, respondents answer 12 questions to assess pain and mobility in relation to the relevant joint. Each item can be scored from 0 (severe problem) to 4 (no problem). Summated scores provide an overview, from 0 (worst) to 48 (best) health statuses [12].

For the Oxford Scores, instructions were adapted to enable usage for retrospective assessment (QR) by including a statement on the timeframe with the following wording; ‘We are interested in finding out about the problems you have had with the hip (knee) on which you have had surgery. Please let us know how you were before your operation’. This kept the wording similar to the instructions for the prospective version use in the National PROMs programme (Q1); ‘We are interested in finding out about the problems you have had with the hip (knee) on which you are about to have surgery’. The tense of individual questions were also altered, e.g. Q1: ‘During the past 4 weeks…How would you describe the pain you usually have from your knee?’ was changed to ‘During the past 4 weeks before your operation…How would you describe the pain you usually had from your knee?’.

The EQ-5D-3L has five questions that investigate the domains of mobility, usual activities, self-care, pain/discomfort and anxiety/depression [13]. For each of these questions, the respondent chooses from three responses indicating the level of their function. A multi-attribute utility score where death and perfect health are represented by 0 and 1 are calculated [14]. Scores less than 0 are considered worse than death and 1 is the maximum score possible. The EQ-VAS (a visual analogue scale) was also included in the questionnaires but this was not included in the analysis of the results, due to missing data and respondents not completing it according to instructions [15].

For the EQ-5D-3L, wording was adapted to provide instructions suitable for retrospective assessment with ‘before your operation’ in place of ‘today’. The full instructions on QR were: ‘By placing a tick in one box in each group below, please indicate which statements best describe your own health state before your operation’. Each statement of individual items was changed to past tense (e.g. ‘I have no problems walking about’ was changed to ‘I had no problems walking about)’.

### Sample size

Sample size was designed to achieve the required degree of precision in the estimation of the ICC. For example, a sample of 200 patients would give a two-sided 95% confidence interval of 0.14 if the ICC was 0.7 (ICC CI 0.62–0.76). Consequently, we selected a total sample of 400 (200 for each procedure), which meant that the width of the CI (0.14) was less than the width of bands used to define categories of agreement (see below). It also provided sufficient statistical power for some sub-group analyses [16, 17].

### Statistical analysis

Agreement between patients’ retrospective and contemporaneous PROMs scores was judged both in terms of absolute agreement and consistency. It was assumed that both time points measure the same construct and should thus be in strong absolute agreement. However, while any systematic differences in recall could reduce absolute agreement, if patients retained their Q1 and QR ranking order, then there would still be consistency in the scores. We therefore also looked at consistency which could be useful from a policy perspective as even if scores lacked absolute agreement but remained consistent, then PROMs retrospective scores would be useful in assessing provider performance. Agreement was categorised as 0–0.20 weak, 0.21–0.40 fair, 0.41–0.60 moderate, 0.61–0.80 strong and 0.81–1 very strong [18].

We calculated separate intraclass correlations for absolute agreement (ICC(A,1)) and consistency (ICC(C,1)) using the definitions given by McGraw and Wong [16], as well as Pearson’s correlation coefficient as a measure of association. The analysis was conducted using Stata version 14 [17]. The ICCs were calculated using repeated measures of analysis of variance (ANOVA) which divides the variance into three components: between-subjects (patients), within-subjects (contemporaneous recall) and error. They are presented with their 95% confidence intervals.

To explore patterns of differences in the contemporary and retrospective score visually, we used a version of the Bland–Altman plot that accounts for trend. Individual differences in scores were plotted against the mean of the two scores, and a regression model was used to calculate the limits of agreement [19]. As neither the contemporaneous nor the retrospective method is assumed to be a gold standard, the mean of the two is the best estimate of the true health status and most appropriate for the *x*-axis [20].

**Fig. 1:**
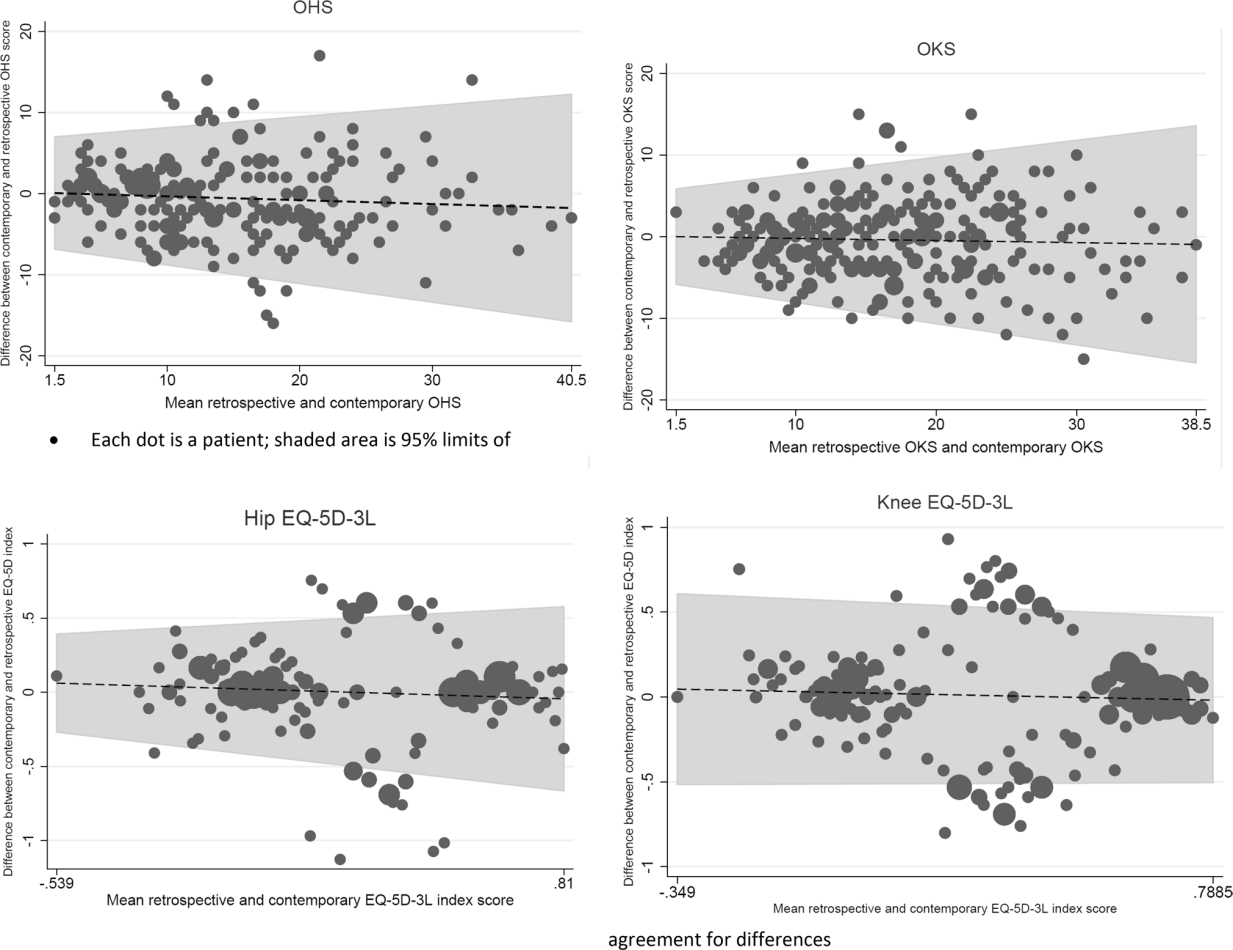
Patterns of differences in contemporary and retrospective PROMs (OHS, OKS and EQ5D) adjusting for trend. Each dot is a patient; shaded area is 95% limits of agreement for differences

Finally, linear regression analysis was conducted to explore whether a patient’s retrospective PROM is able to predict their contemporary PROM, judged from differences in their predicted (based on retrospective) and contemporary PROM (mean absolute error). Scatterplots of contemporary score (*y*-axis) against the retrospective score (*x*-axis) are shown in Fig. 1, along with the mean predicted score (linear fit) and 95% confidence intervals. The wider lines show the 95% confidence intervals around individual predictions, taking into account the residual variation in individual scores.

The influence on the relationship between retrospective and contemporary PROMs of two patient characteristics (age and social-economic status) and one logistical (length of time between the two data collection points) was explored using linear regression analysis; ICCs were also calculated for age subgroups.

## Results

### Patient characteristics

The required sample size of 400 in total was exceeded. Of the 406 hip replacement patients who had completed a Q1 and were invited to complete a QR, 244 (60%) did so. Equivalent figures for knee replacement were 276 out of 486 (57%). It was not possible to link data from the two questionnaires for some patients (20 hip; 16 knee) and the disease-specific PROM was not fully completed by some patients (20 hip; 21 knee) (Appendix). This left 204 hip and 239 knee patients for the analysis.

The sample was broadly similar to the population of patients completing pre-operative PROM questionnaires in 2009–2010, the latest year for which published data exist [21, 22]. There were some small differences (Table 1). The hip replacement sample was slightly older (mean age 69.1 vs. 67.7 years) and more likely to be female (67 vs. 61%), and to live alone (34 vs. 28%). The knee patients were also more likely to live alone (29 vs. 25%). For both operations, patients reported having more severe conditions (mean OHS 15.1 vs. 18.2; mean OKS 17.4 vs. 19.3; knee symptoms for over 5 years 55 vs. 44%). This may reflect selection bias in the sample or a change between 2009/2010 and 2016 in the severity of patients’ conditions.

**Table 1:** Characteristics of samples compared with population of patients (2009–2010) [21, 22]

Characteristic	Hip replacement		Knee replacement	
	Sample	Population	Sample	Population
Sex: female (%)	136 (67)	(61)	152 (61)	(57)
Age—mean (SD)	69.1 (12.4)	67.7	68.7 (9.0)	68.7
Living arrangements (%)				
With family/friends	65	72	70	75
Alone	34	28	29	25
Other	1	0	1	0
Duration of symptoms (%)				
0–5 year	79	81	45	57
> 5 years	21	19	55	44
Primary operation (%)	91	90	90	92
Disease-specific PROM Q1—mean (SD)	15.1 (8.7)	18.2	17.4 (8.2)	19.3
EQ-5D-3L Q1—mean (SD)	0.24 (0.33)	0.36	0.35 (0.32)	0.43

While most patients (75%) completed their QR within 50 days of having completed the contemporary Q1, for 3% it was over 3 months (due to delays in surgery following their pre-operative assessment). The median length of time was 30 days (IQR 14–54 days).

### Comparison between retrospective and contemporary PROMs

The mean difference between retrospective and contemporary scores was small for all PROMs and both operations (Table 2). The direction of the difference was consistent: patients reported slightly lower scores (worse health) in the retrospective questionnaire compared to the contemporary reports. However, none of the differences were statistically significant.

**Table 2:** Agreement between contemporary and retrospective PROMs

PROM	Mean Q1:QR	Mean difference (95% CI)	*p* value	ICC absolute agreement (95% CI)	ICC consistency (95% CI)
OHS	15.07:14.56	0.51 (− 0.19 to 1.23)	0.15	0.82 (0.77–0.86)	0.82 (0.77–0.86)
OKS	17.36:17.02	0.34 (− 0.30 to 0.98)	0.29	0.82 (0.77–0.85)	0.82 (0.77–0.85)
Hip EQ-5D	0.24:0.22	0.02 (− 0.02 to 0.06)	0.3	0.62 (0.53–0.69)	0.62 (0.56–0.69)
Knee EQ-5D	0.35:0.32	0.03(− 0.01 to 0.07)	0.16	0.60 (0.51–0.67)	0.60 (0.51–0.67)

**Fig. 2:**
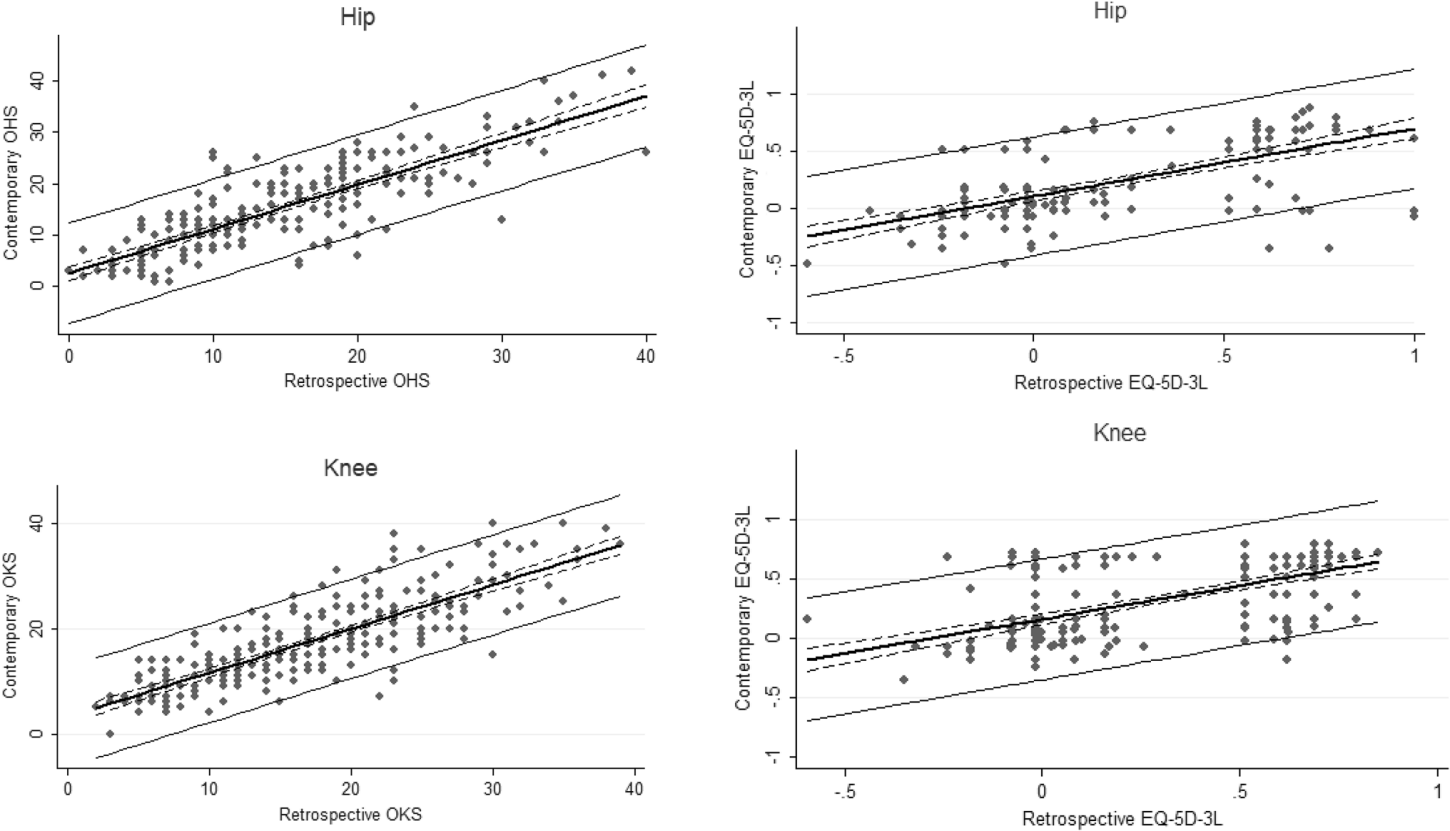
Contemporary PROM by retrospective PROM linear regression with 95% intervals for individual (solid line) and group (dotted line) contemporary PROMs predictions. Dots represent actual PROM scores, and the solid line the predicted contemporary PROMs scores with 95% intervals for individual and group predictions

Absolute agreement and consistency were very strong for both disease-specific PROMs. Agreement on the EQ-5D-3L was also strong, although weaker than for the disease-specific PROM. The level of agreement was consistent across the range of severity of pre-operative health (i.e. there was little systematic bias) as shown by the flat trend lines (Fig. 2). The clustering seen for the EQ-5D-3L results from there being only three possible levels of response to each item and the way one dimension, pain/discomfort, is weighted heavily in the index score. Therefore, patients who shifted in their level in the pain dimension resulted in a more marked change in their index score, while the average of their two scores was in the middle (see Fig. 1 EQ-5D). In contrast, there was greater concordance between retrospective and contemporary scores in patients who reported either no or extreme pain/discomfort and who did not shift their responses (with their average of their two score remaining at one extreme or the other, i.e. responses seen in the clusters to the most left and furthest right on the horizontal axis).

### Prediction of contemporary using retrospective PROMs scores

Patients’ retrospective PROMs were able to predict contemporary scores for all three PROMs. The mean absolute error for the prediction model were 3.89 (Q1 SD 8.7) and 3.86(Q1 SD 8.2) for the Oxford Hip and Knee scores and 0.20 and 0.21 for generic EQ-5D scores at the individual level (Table 3). At the group level, this would translate into an even smaller error. The 95% confidence intervals for the mean predicted score (group prediction) is extremely narrow (Fig. 2).

**Table 3:** Retrospective scores as a predictor of contemporary PROMs

PROM	Pearson correlation coefficient (*r*)	Mean absolute error	Coefficient B (95% CI)
Oxford Hip Score	0.82	3.89	0.85 (0.77–0.94)
Oxford Knee Score	0.82	3.68	0.84 (0.76–0.91)
Hip EQ-5D	0.58	0.20	0.62 (0.50–0.73)
Knee EQ-5D	0.56	0.21	0.59 (0.48–0.70)

### Influences on relationship between contemporary and retrospective PROMs

Agreement between the retrospective and contemporary PROM was strong or very strong across the age range, although slightly weaker with increasing age. For hip patients, the ICC declined from 0.88 for those aged 60 years or younger to 0.78 for those over 75 years (*p* value < 0.05). The difference for knee patients was less (ICC 0.80 vs. 0.78). There was no evidence of any systematic differences in the magnitude and the direction of recall with patients’ age as well as socio-economic status for both Oxford Hip and Oxford Knee Scores. There was some evidence of a slight systematic difference with patients’ age on EQ-5D-3L for knee patients (Table 4).

**Table 4:** Mean difference and adjusted mean difference between retrospective and contemporary PROMs by patients’ socio-economic status (SES) and age

Patient characteristic	Hip replacement (OHS)					Knee replacement (OKS)				
	*n* (%)	Mean Q1:QR	Mean difference	Adjusted difference	*p* value	*n* (%)	Mean Q1-QR	Mean difference	Adjusted difference	*p* value
SES (quintiles)				*Adjusted by age					*Adjusted by age	
1 (Least deprived)	15 (7.4)	15.8–17.0	− 1.13	− 0.72 (− 3.64 to 2.20)		26 (10.6)	18.1–20.5	− 2.35	− 1.74 (− 4.18 to 0.65)	
2	36 (17.7)	15.1–13.3	1.89	2.14 (0.01 to 4.29)		43 (17.6)	19.4–18.3	1.02	1.46 (− 0.60 to 3.53)	
3	57 (27.9)	14.8–14.9	− 0.14	Reference group	0.16	48 (19.6)	16.1–16.5	− 0.31	Reference group	0.10
4	54 (26.5)	16.4–15.2	1.25	1.44 (− 0.44 to 3.34)		69 (28.2)	17.4–16.9	0.52	0.85 (− 1.92 to 1.75)	
5 (Most deprived)	42 (20.6)	13.5–13.4	0.14	0.26 (− 1.76 to 2.28)		58 (23.7)	14.7–15.1	− 0.36	− 0.81 (− 2.73 to 1.11)	
Age (years)				*Adjusted by SES					*Adjusted by SES	
≤ 60	49 (24)	16.3–15.8	0.53	− 0.27 (− 2.09 to 1.53)		43 (18)	16.9–15.3	1.62	2.00 (0.29 to 3.73)	
61–75	85 (42)	15.2–14.2	1.06	Reference group	0.43	137 (57)	17.9–18.6	− 0.72	Reference group	0.07
> 75	70 (34)	14.1–14.1	0.14	− 1.05 (− 2.67 to 0.56)		59 (25)	14.9–14.7	0.23	− 0.84 (− 0.67 to 2.37)	

Patient characteristic	Hip replacement (EQ-5D-3L index)					Knee replacement (EQ-5D-3L index)				
	*n* (%)	Mean Q1:QR	Mean difference	Adjusted difference	*p* value	*n* (%)	Mean Q1-QR	Mean difference	Adjusted difference	*p* value
SES (quintiles)				*Adjusted by age					*Adjusted by age	
1 (Least deprived)	19 (8.6)	0.17–0.29	− 0.12	− 0.08 (− 0.25 to 0.09)		28 (11)	0.33–0.41	− 0.07	− 0.08(− 0.22 to 0.07)	
2	40 (18.2)	0.22–0.17	0.05	0.08 (− 0.05 to 0.21)		45 (17.7)	0.39–0.39	0	− 0.02 (− 0.15 to 0.10)	
3	62 (28.2)	0.22–0.25	− 0.02	Reference group	0.39	50 (19.7)	0.32–0.29	0.02	Reference group	0.12
4	55(25)	0.28–0.28	− 0.002	0.03 (− 0.09 to 0.14)		71 (28.0)	0.31–0.33	− 0.02	− 0.05 (− 0.16 to 0.06)	
5 (Most deprived)	44 (20)	0.17–0.14	0.03	0.04 (− 0.08 to 0.17)		60 (23.6)	0.32–0.23	− 0.09	0.06 (− 0.06 to 0.18)	
Age (years)				*Adjusted by SES					*Adjusted by SES	
≤ 60	51 (23.2)	0.30–0.29	0.01	− 0.02 (− 0.13 to 0.09)		46 (18.1)	0.26–0.18	0.07	0.09 (− 0.01 to 0.20)	
61–75	95 (43.2)	0.24–0.21	0.03	Reference group	0.23	147(57.9)	0.35–0.39	− 0.04	Reference group	0.04
> 75	74 (33.6)	0.14–0.20	− 0.06	− 0.08 (− 0.18 to 0.01)		61 (24)	0.34–0.26	0.08	0.10 (0.01 to 0.20)	

*OHS* Oxford Hip Score, *OKS* Oxford Knee Score

The difference in mean contemporary and retrospective scores was not associated with the time interval between Q1 and QR. The difference in Oxford Knee Score decreased by 0.013 (95% CI − 0.03 to 0.007) and knee EQ-5D-3L score decreased 0.0003 (95% CI − 0.001 to 0.0007). The difference for Oxford Hip Score increased by 0.006 (95% CI − 0.01 to 0.02) per day, and the hip EQ-5D-3L score increased by 0.0001 (− 0.0009 to 0.001) per additional day.

## Discussion

### Main findings

In representative samples of patients undergoing elective hip or knee replacement, their retrospective assessment of their pre-operative health status was similar to their contemporaneous reports. Although patients tended to recall their health as being slightly worse than reported at the time across all measures, the differences were small and none was statistically significant. This could result in a slightly higher estimation of the benefits of surgery. The level of agreement between contemporary and recalled PROM scores was very strong for the disease-specific ones, and strong for the generic PROM.

The strength of agreement was consistent regardless of the severity of a patient’s primary condition. In addition, two social characteristics of patients, their age and their socio-economic status, had little or no significant influence on the relationship between retrospective and contemporary reports. It was also apparent that mean retrospective PROMs for groups of patients could reliably predict what mean contemporary reports of PROMs would have been.

### Comparison to existing studies

These results confirm the findings of the four published studies which also found strong and very strong agreement between retrospective and contemporary PROMs which used continuous rather than categorical data [8, 23–25]. These previous studies also found that agreement for disease-specific PROMs was stronger than for generic PROMs. One explanation for this is that generic measures tend to have a more restricted range of responses, leading to greater homogeneity (smaller between-patient variability) in scores. ICCs define agreement between scores (within patients) in relative terms, so smaller population variation in scores will necessarily limit the strength of agreement.

These results suggest the main factors that may influence the differences between contemporary and retrospective reports, namely recall bias and response shift (a change in perception that can occur when circumstances change), did not have a significant influence. This may partly reflect the short time interval between measurements. Recall bias may arise when details of events go unnoticed and are not stored; new information is added to stored memories altering the details; and, over time, events are systematically distorted [26]. Such bias is influenced by the time between the event and its assessment: the longer the interval, the higher the probability of recall bias [27].

The lack of association between agreement and the length of the recall time in our results suggests that recall bias was minimal. It may be the case, as implicit theories of memory suggest, that the act of asking people to recall how they were before their surgery provided an anchor of their pre-surgical condition and hence formed the basis for stable recollection [28]. There is also a possibility that the exposure to a prior PROMs questionnaire could have aided recall. However, as an event in the patient’s life, this is likely to pale in comparison with the subsequent hospital admission and operation in terms of a ‘significant event’ in the process of aiding the anchoring and assisting recollection of the patient’s prior health.

The weaker agreement observed with the EQ-5D-3L is consistent with two previous studies that showed only moderate agreement when using PROMs with categorical data rather than continuous data [6, 7]. Lingard et al. [7] found this when items were not evenly distributed (i.e. when responses are clustered to at the severe end of the scales, e.g. severe pain and limited function).

As in this study, two previous studies observed the strength of agreement was high across age groups but decreased slightly with increasing age: OHS ICC for under 65 years 0.95 versus 0.85 for those older [8]; Western Ontario & McMaster Osteoarthritis Index for knee pain under 75 years 0.57 versus 0.47 for those older [7].

### Strengths and limitations

This is the second largest such study ever undertaken, in addition to assessing agreement with ICCs which allowed differentiation between perfect agreement, systematic and random bias [29]. Bland–Altman plots [20] have provided a visual display of systematic bias or differences in relation to the scales of the PROMs providing an additional layer of understanding.

The one potential limitation concerns the representativeness of the sample who participated. Although they were broadly similar to the population of patients undergoing arthroplasty in England, they may have differed as regards some other unmeasured variables. It is possible that people who agreed to participate were more consistent in their recalled reports than the general population of patients.

### Implications

These findings support the use of retrospective PROMs to obtain a baseline assessment of health status when contemporary collection is not feasible such as with emergency hospital admissions. In addition, retrospective collection offers an alternative even when contemporary is possible, an option that could not only facilitate higher participation rates but also lower the cost of data collection.

While this study has demonstrated the feasibility of collecting retrospective PROMs in patients who are recovering from an elective procedure (and who have already agreed to participate in a pre-operative contemporary report), research is now needed to determine the feasibility in emergency admissions. The latter have experienced an unexpected, sudden episode of illness and may still be unwell some days later. Whether collection of retrospective PROMs is feasible needs to be investigated.
